# P-130. A Case-Control Study of Post-Infectious Sequelae Following a Shiga Toxin-Producing *Escherichia coli* (STEC) Outbreak at a U.S. Marine Corps Recruit Depot

**DOI:** 10.1093/ofid/ofae631.335

**Published:** 2025-01-29

**Authors:** Michelle Kautz, Derek Larson, Terrel Sanders, Hsing-Chuan Hsieh, Jennifer McAnany, Zeina G Khodr, Sheila F Castaneda, Ryan C Maves, Tahaniyat Lalani

**Affiliations:** Naval Medical Center San Diego, Uniformed Services University Department of Medicine, San Diego, California; Fort Belvoir Community Hospital and Uniformed Services University, Fort Belvoir, Virginia; Naval Medical Research Unit Three Ghana Detachment, MARION, South Carolina; Infectious Disease Clinical Research Program, Bethesda, Maryland; NHRC/Leidos, San Diego, California; Leidos, Inc ; Naval Health Research Center, San Diego, California; Naval Health Research Center, San Diego, California; Wake Forest University School of Medicine, Winston-Salem, North Carolina; Naval Medical Center Portsmouth, Portsmouth, Virginia

## Abstract

**Background:**

In November 2017, an outbreak of Shiga toxin-producing *Escherichia coli* (STEC) O157:H7 and O26 occurred among male recruits at a United States Marine Corps Recruit Depot, San Diego, CA. We sought to evaluate the 6-year risk of hypertension (HTN), chronic kidney disease (CKD), and irritable bowel syndrome (IBS) following STEC infection by conducting a retrospective, case-control study utilizing medical record data.

**Table 1.**
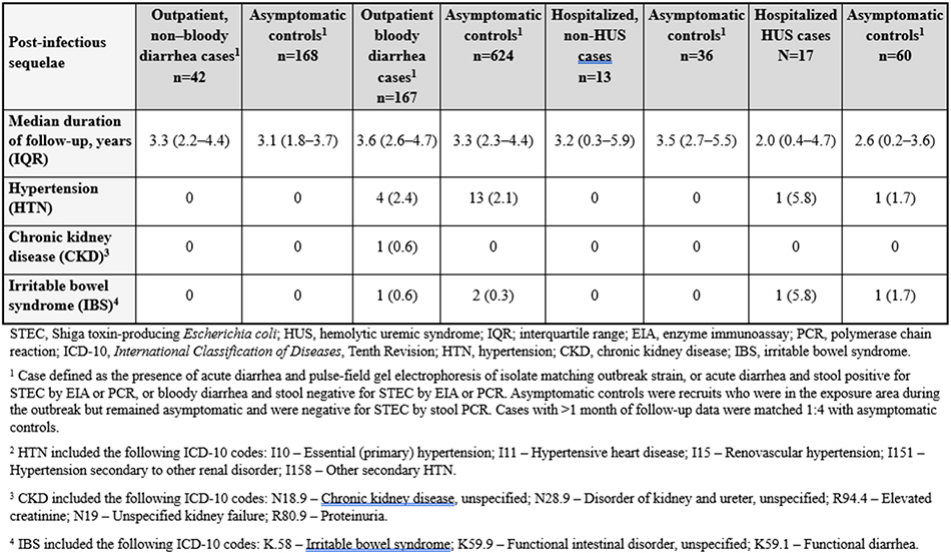
Proportion of STEC cases and matched asymptomatic controls with post-infectious sequelae following November 2017 outbreak

**Methods:**

Data from the outbreak investigation were used to identify cases and asymptomatic controls. Asymptomatic controls were recruits who were in the exposure area during the outbreak but remained asymptomatic and were negative for STEC by stool PCR. Cases were matched 1:4 with asymptomatic controls based on the duration of follow-up available in the Military Health System Data Repository (MDR). Controls could be matched to > 1 case. Post-infectious outcomes were assessed using ICD-10 diagnostic codes in the MDR.

**Results:**

239 cases were identified of whom 192 (80%) developed bloody diarrhea, 30 (13%) were hospitalized, and 17 (7%) developed hemolytic uremic syndrome (HUS). The *stx-2* gene was detected in 39% of PCR positive cases. The median age of recruits was 19 years, and the median duration of MDR follow-up for cases and controls was 3.7 years (IQR: 2.9-5.4). There was no significant difference in the proportion of cases and controls with post-infectious sequelae (Table 1). Resolution of renal dysfunction was noted within 3 months after discharge for all HUS cases, and only 1 case remained hypertensive following discharge. There was no significant difference in the proportion of bloody diarrhea and HUS cases that developed IBS compared to controls. A limitation of the analyses was the short duration of MDR follow-up available for 6/30 hospitalized cases due to military separation after hospital discharge.

**Conclusion:**

STEC infection in this cohort of young military recruits was associated with a good prognosis, even among cases of STEC-associated HUS, with resolution of renal dysfunction and no increase in incidence of IBS or HTN compared with asymptomatic controls. The findings suggest that STEC infection in young adults who recover from acute illness is not associated with a higher likelihood of post-infectious sequelae.

**Disclosures:**

**Ryan C. Maves, MD**, AiCuris: Grant/Research Support|Biotest: Grant/Research Support|GeoVax: Grant/Research Support|Shionogi: Advisor/Consultant|Shionogi: Honoraria|Sound Pharmaceuticals: Grant/Research Support

